# A new species of *Thinouia* (Paullinieae, Sapindaceae) from the Amazon and its phylogenetic placement

**DOI:** 10.3897/phytokeys.165.57341

**Published:** 2020-10-28

**Authors:** Herison Medeiros, Jenifer de Carvalho Lopes, Pedro Acevedo-Rodríguez, Rafaela Campostrini Forzza

**Affiliations:** 1 Universidade de São Paulo, Instituto de Biociências, Departamento de Botânica, Rua do Matão, 277, 05508-090 São Paulo, SP, Brazil Jardim Botânico do Rio de Janeiro Rio de Janeiro Brazil; 2 Department of Botany, MRC-166 Smithsonian Institution, P.O. Box 37012, Washington D.C. 20013-7012, USA Universidade de São Paulo São Paulo Brazil; 3 Jardim Botânico do Rio de Janeiro, Pacheco Leão, 915, 22460-030, Rio de Janeiro, Rio de Janeiro, Brazil Department of Botany Washington United States of America

**Keywords:** *Allosanthus*, Amazonia, Brazil, lianas, neotropical biodiversity, Paulliniodae, Paullinieae, Sapindales, Sapindaceae, taxonomy, *Thinouia*

## Abstract

*Thinouia* is a Neotropical genus of lianas with approximately 12 species and is the only genus in tribe Paullinieae with actinomorphic flowers. During a taxonomic revision of the genus and fieldwork in south-western Amazonia, we found a new species that appears similar to *Thinouiatrifoliata* (ex *Allosanthus*) because of its racemiform inflorescence. However, before describing the new species, we had to confirm that *Allosanthus* was congeneric with *Thinouia* so we could place the new species in the correct genus. The results of the phylogenetic analysis, based on molecular data (*trnL* intron and ITS sequences), show that *Allosanthus* should be included in *Thinouia*. Thus, the new taxon is described here as *Thinouiacazumbensis***sp. nov**. The new species is described, illustrated and phylogenetic trees showing relationships within supertribe Paulliniodae and *Thinouia* and the congeneric *Allosanthus* are given.

## Introduction

*Thinouia* is a neotropical genus of lianas that includes around 12 species, of which *T.myriantha* Planch. & Triana is widely distributed, including records from Mexico, Central America and northern South America (Ferrucci and Somner 2008; Acevedo-Rodríguez et al. 2011). The remaining species are distributed in Brazil, Bolivia, Paraguay and Peru, except for *T.tomocarpa* Standl. which is restricted to Mexico, Belize and Guatemala. Most *Thinouia* species occur in rainforest; a few species occur in savannah (BFG 2015).

*Thinouia* was proposed by Triana and Planchon (1862). It is characterised by the presence of umbelliform and racemiform thyrses, actinomorphic flowers with marginal or bifid petal appendages, an annular disc and schizocarpic fruits that split into three mericarps, each with a distal wing (Ferrucci and Somner 2008; Acevedo-Rodríguez et al. 2017).

Molecular phylogenetic studies show that *Thinouia* is a monophyletic group in Sapindaceae. In the most recent phylogenetic study, tribe Paullinieae (i.e. *Cardiospermum*, *Lophostigma*, *Paullinia*, *Serjania*, *Thinouia* and *Urvillea*) is a well-supported clade with *Thinouia* sister to the remaining genera (Acevedo-Rodríguez et al. 2017). In the same work, the monospecific genus *Allosanthus* (*A.trifoliatus* Radlk.) was maintained as a synonym of *Thinouia*, based on morphological characters. The only differentiating character (i.e. a racemiform inflorescence) was not considered worthy of generic recognition (Acevedo-Rodríguez et al. 2011, 2017).

During a taxonomic revision of the genus and fieldwork in south-western Amazonia, we found a new species of *Thinouia* that is similar to *Thinouiatrifoliata* (Radlk.) Acev.-Rodr. & Ferrucci because of its racemiform inflorescence. Since we now have high-quality DNA material for the taxa previously assigned to *Allosanthus*, we re-analysed the placement of *Allosanthus* within *Thinouia* and further tested the monophyly of *Thinouias.l.*, which revealed the correct position of the new species.

## Material and methods

### Plant material

We collected the new species in Reserva Extrativista do Cazumbá-Iracema in Sena Madureira, Acre, Brazil. The collection was pressed and dried for vouchers, leaves were collected in silica gel for DNA extraction and reproductive structures were fixed in 70% alcohol for morphological analyses, which were performed using a stereomicroscope. The morphological structures were described using the terminology in Radford et al. (1974) and Weberling (1989). The herbarium abbreviations cited in the text follow Thiers (2020, cont. upd.).

### Phylogenetic analysis

The phylogenetic analysis included the same taxa and molecular markers of Acevedo-Rodríguez et al. (2017), 93 taxa, plastid marker trnL intron and nuclear ribosomal internal transcribed spacer, ITS. Six samples (*Allosanthus* sp., *Allosanthustrifoliatus*, *Thinouiamucronata*, *T.myriantha*, *T.obliqua* and *Thinouia* sp.), including the new species, were added to the analysis, using the same molecular markers. For these additional taxa, approximately 60 mg of leaf tissue were pulverised with Tissuelyzer (Qiagen, Duesseldorf, Germany) for 3 min at 60 hz. The DNA extraction used the DNA NucleoSpin Plant II kit (Machery-Nagel, GmbH & Co. KG, Dueren, Germany) following the manufacturer’s protocol. Primers and the PCR amplification were used, as described in Acevedo-Rodríguez et al. (2017). Products were purified and sequenced by Macrogen (Seoul, South Korea). All sequences, vouchers and GenBank accession numbers are summarised in Appendix I.

The alignments were performed using MAFFT (Katoh et al. 2002) using the default parameters implemented in Geneious 2020.0.5 (Kearse et al. 2012). Poorly-aligned regions were removed and adjusted manually. We used jModelTest 2.0 (Guindon et al. 2010; Darriba et al. 2012) and the Akaike Information Criterion (AIC) to select the best-fit model of nucleotide substitution for each dataset. The GTR+I+G was selected as the best model for the ITS dataset, whereas the GTR+G was selected as the best model for the trnL dataset. Bayesian Inference (BI) analyses were conducted using MrBayes 3.2.2 (Ronquist et al. 2012) in the online CIPRES Science Gateway interface (Miller et al. 2015) with four Markov Chain Monte Carlo (MCMC) runs using a random starting tree and 10 million generations, with a sampling frequency of one every 1000 generations. We used Tracer 1.7 (Rambaut et al. 2018) to check for convergence of the MCMC and to check for stationarity. We discarded 25% of the trees as burn-in.

Phylogenetic trees were plotted and built inside the R environment (R Core Team 2020), version 3.6.2, using the packages ggplot2 (Wickham et al. 2020), ggtree (Yu et al. 2017; Yu and Lam 2020) and cowplot (Wilke 2019).

## Results

### Phylogenetic results

**Figure 1.**
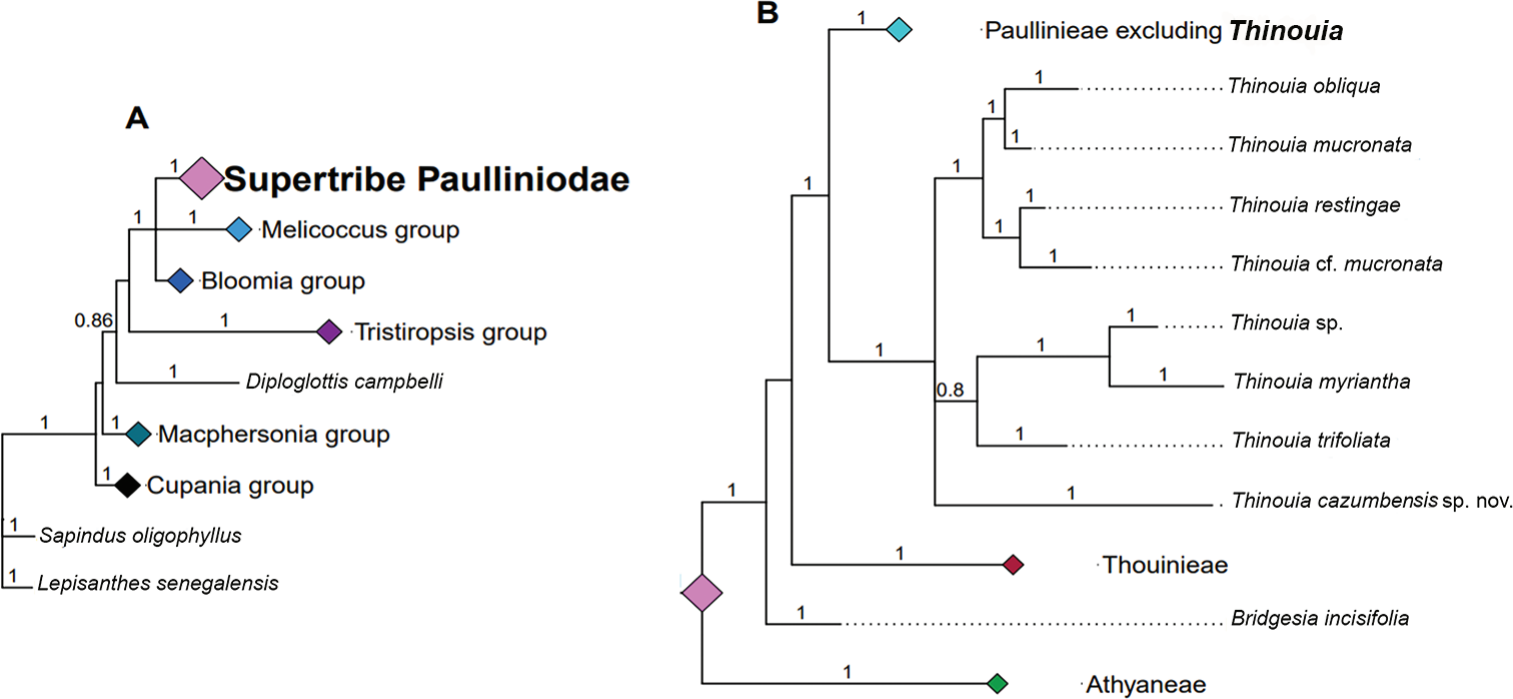
**A** bayesian 50% majority-rule consensus tree from a Bayesian analysis of the combined, two-marker dataset for Paulliniodae and outgroups **B** relationships of *Thinouia* and the congeneric *Allosanthus* [(=*Thinouiatrifoliata* (Radlk.) Acev.-Rodr. & Ferrucci], including the newly-described *Thinouiacazumbensis* sp. nov. Bayesian posterior probability values are indicated above the branches.

The ITS dataset included 99 terminals and 876 bp, the trnL dataset included 99 terminals and 727 bp and the combined dataset included 99 terminals and 1604 bp. Phylogenetic trees from the analyses of the combined dataset showed high posterior probability values (PP > 0.8). Only the topology from the combined analysis is described here (Fig. 1). Separate analyses of each locus did not reveal any strong groupings that would indicate incongruences.

Supertribe Paulliniodae is strongly supported as monophyletic (Fig. 1 A, PP = 1.0). The tribe Paullinieae is also strongly supported as monophyletic (PP = 1.0) and the genus *Thinouia* (including *Allosanthus*) is recovered as the clade, sister to the remaining genera of the tribe Paullineae (PP = 1.0). *Thinouia* species are grouped in two main clades that are in a polytomy with the new species *Thinouiacazumbensis*. The first clade (PP = 1.0) includes *Thinouiaobliqua*, *T.mucronata*, *T.restingae* and T.cf.mucronata species. The second one (PP = 0.8) includes *Thinouia* sp., *T.myriantha* and *T.trifoliata* (= *Allosanthustrifoliatus* Radlk.) (Fig. 1).

### Taxonomic treatment

#### Thinouia cazumbensis

Taxon classificationPlantaeSapindalesSapindaceae

Medeiros sp. nov.

767D9AA5-E898-5F21-BB0B-18F0CBADF2B7

urn:lsid:ipni.org:names:77212573-1

Figure 2

##### Diagnosis.

The new species differs from *Thinouiatrifoliata* by the 5-lobed floral disc, fruits with trichomes and basal petal appendages smaller than the petals, versus annular disc, glabrous fruits and marginal petal appendages larger than the petals.

**Figure 2.**
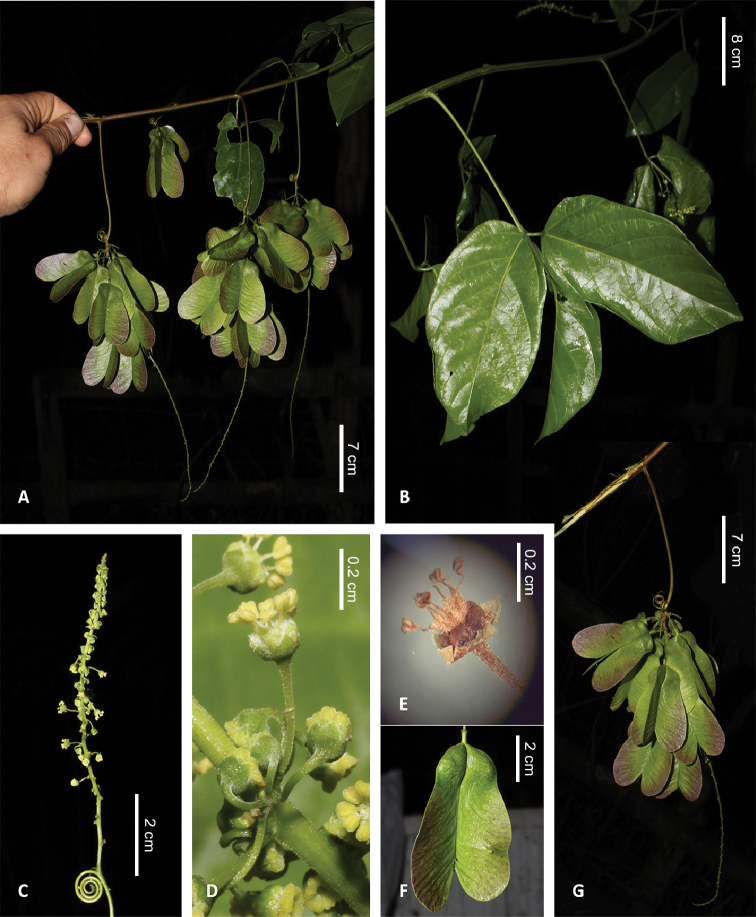
*Thinouiacazumbensis***A** fruiting branch **B** detail of leaf, abaxial view **C** racemiform inflorescence with a pair of basal tendrils **D** detail of inflorescence (cincinnus) **E** flower with removed petals showing a 5-lobed nectary disc **F** detail of fruit **G** infructescence (**A–G**) from H. Medeiros 3401 (RB). Photos by H. Medeiros.

##### Type.

Brazil. Acre. Sena Madureira. Reserva Extrativista do Cazumbá-Iracema, Núcleo Cazumbá, castanhal coletivo, floresta ombrófila aberta com bambu, 9°8'30"S, 68°56'23"W, 20 Jul 2018, *H. Medeiros, M. Silveira & E.M. Soares 3401*, (holotype RB!; isotypes: INPA!, SPF!, UFACPZ!, US!).

##### Description.

Tendrilled liana 6–8 m long; stem puberulent, with yellowish to whitish indumentum, lenticellate; cross section simple, cylindrical. Leaves trifoliolate; stipules ca. 2 mm long, hirsute-tomentose, linear triangular to lanceolate; petiole 2–8.5 cm long, canaliculate; petiolules of lateral leaflets 0.2–0.8 cm long; leaflets 7–14 × 3–9 cm, oblong to ovate- rhomboidal, apex acute, mucronate, margins entire to dentate-serrate, with 2–4 teeth reduced to inconspicuous glands, ciliate, base truncate, rounded to obtuse, sometimes cuneate on the distal leaflet, glabrous on both surfaces, domatia sometimes in the axils of abaxial secondary veins. Thyrses axillary, racemiform, ca. 8.5–16 cm long, peduncle 1.1–2.8 cm long, rachis of 7.5–16 cm long; numerous cincinnus, sessile. Flowers ca. 2 mm long, pedicel ca. 0.5 mm long; sepals 5, ca. 1 mm long, fused at the base, lobes ovate, acute, glabrous and with prominent veins on the internal surface, external surface villous; petals 5, ca. 1.5 mm long, obovate, obtuse, clawed, villous on the central part and margins, the rest glabrous; petal appendages rudimentary, bifid, smaller than the petals, basally adnate, villous; nectary disc glabrous, 5-lobed, lobes ca. 1 mm long; staminate flower: stamens 8, 1.5 mm long, filaments villous for more than half of their length, anthers glabrous, pistillode ca. 1.5 mm long; pistillate flower: staminodes ca. 1 mm long, pistil ca. 1.5 mm long, style 0.5 mm long, with 3 stigmas, ovary ca. 1 mm long. Fruits ovate, chartaceous, 5–5.5 × 2–2.3 cm; cocci slightly inflated, 1.2–1.4 × 1.1–1.4 cm, including the ca. 2–3 mm long stipe constricted at junction with wing; epicarp densely strigose (simple trichomes of same length) on cocci, strigose on wings; endocarp glabrous. Seeds trigonous ovoid, ca. 6 × 4 mm, basally attached, glabrous, mature embryo not observed.

*Thinouiacazumbensis* is differentiated from most species of *Thinouia* by the thyrses axillary, racemiform (Fig. 2A, C) and the 5-lobed nectary disc, a character that is unique and for the first time recorded in the genus (Fig. 2E). The lobed nectary disc within *Thinouia* should be further investigated through morpho-anatomical studies to understand how nectaries evolved within the genus.

##### Distribution and ecology.

**Figure 3.**
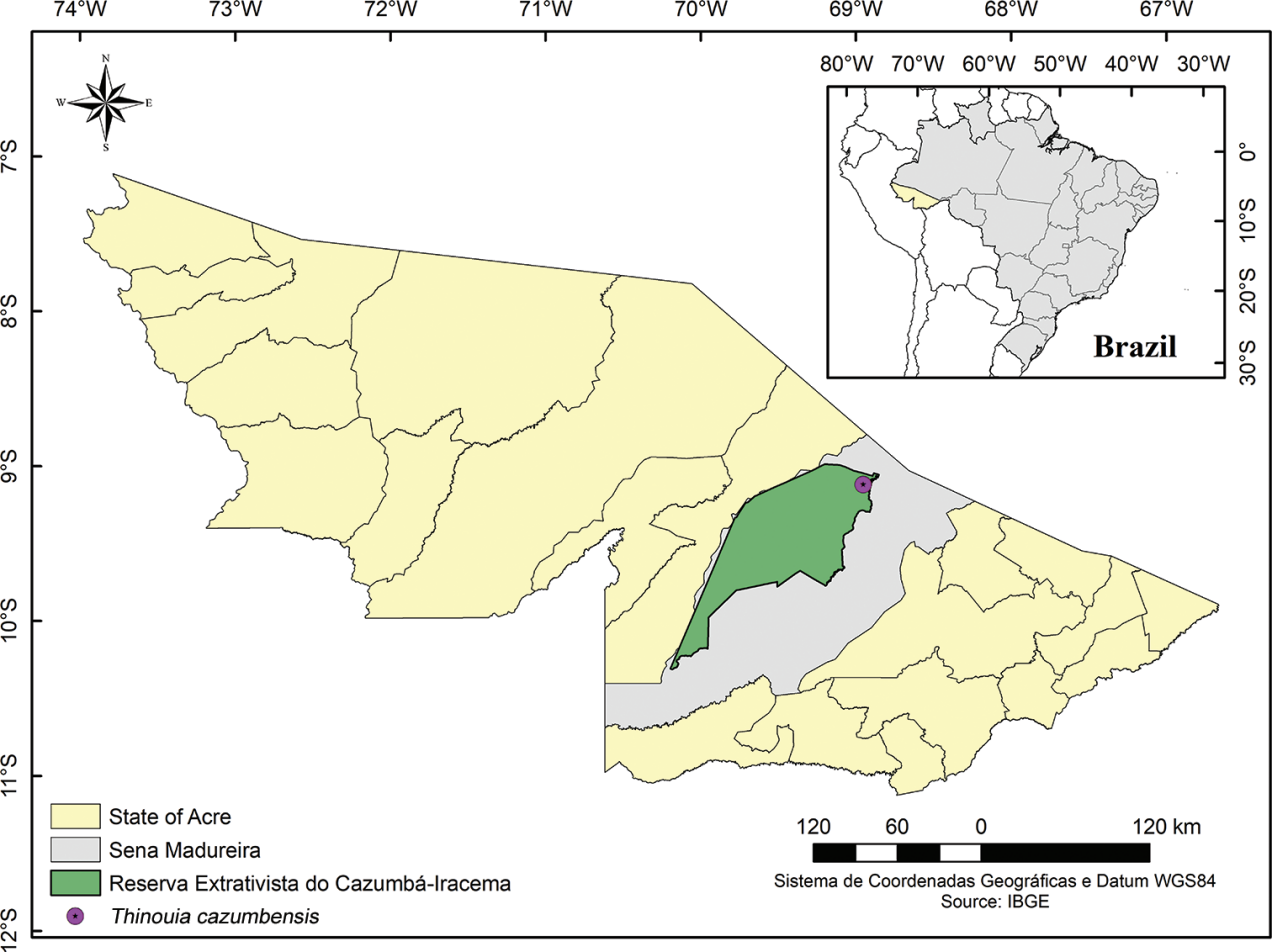
Geographic distribution of *Thinouiacazumbensis*.

*Thinouiacazumbensis* is known only from the Reserva Extrativista do Cazumbá-Iracema (Fig. 3) where it is an infrequent liana that reaches the canopy of the open rainforest with abundant bamboo (*Guadua* spp.) (Silveira 2005).

##### Phenology.

Collected in flower and fruit during July.

##### Etymology.

The epithet *cazumbensis* refers to Reserva Extrativista do Cazumbá-Iracema, where the species was collected. In the 1980s, local rubber tappers and extractivists fought against the area becoming a rural settlement and on 19 September 2002 succeeded in getting the area designated as a conservation unit (ICMBio 2007). Situated in the State of Acre between the municipalities of Sena Madureira and Manoel Urbano, the Reserva Extrativista do Cazumbá-Iracema covers an area of 750,794.70 hectares of the Western Amazon Corridor, one of the seven major ecological corridors proposed for Brazil (Ricardo and Lima 2004).

##### Conservation status.

The species is only known from one locality in Acre and is categorised as Data Deficient (DD) according to IUCN (2019). Further field studies are needed to evaluate its conservation status more accurately.

## Discussion

The broader relationships that we recovered within supertribe Paulliniodae largely agree with those in Acevedo-Rodríguez et al. (2017). Additionally, with the inclusion of new sequences of *Thinouia* in this study merged with sequence data from Acevedo-Rodríguez et al. (2017), our results recovered the same clades in tribe Paullinieae, where *Thinouia* forms a clade that is the earliest diverging lineage. Therefore, our phylogenetic results reinforce including *Allosanthus* in *Thinouia* as proposed by Acevedo-Rodriguez et al. (2011), based on morphological characters. The only differentiating morphological character (i.e. the racemiform inflorescence) was not considered worthy of generic recognition by Acevedo-Rodriguez et al. (2011, 2017) and the molecular data in the present study corroborate this conclusion. The position of the new species as a member of *Thinouia* is strongly supported albeit its relationship to other species is not fully resolved, perhaps because of our limited sampling of *Thinouia* or because only two markers have been sequenced.

## Conclusion

*Thinouiacazumbensis* is supported as a distinct taxon, based on morphological and molecular sequence data. Its position within the genus is still undetermined, highlighting the need for in-depth taxonomic studies on this genus. Ongoing systematics studies, based on molecular and morphological analyses of *Thinouia*, should provide additional insights into the evolution and biogeographic history of this neotropical genus (H. Medeiros et al. in prep.).

## Supplementary Material

XML Treatment for Thinouia cazumbensis

## Appendix I

Voucher and GenBank information for the taxa included in the phylogenetic analyses. Listed as: taxon, collection, herbarium, place of origin and GenBank accession numbers (ITS, trnL intron). Herbarium acronyms follow Index Herbariorum (Thiers, continuously updated).

*Allophylastrumfrutescens* Acev.-Rodr., Lima 812 (K), Brazil, KX584885, KX584982. *Allophylusabyssinicus* (Hochst.) Radlk., Desissa & Binggeli DD-318 (MO), Ethiopia, KX584886, KX584983. *Allophylusafricanus* P. Beauv., Balkwill 4206 (MO), South Africa, KX584887, KX584984. *Allophylusarboreus* Choux, Wohlhauser & Stiefle 60072 (MO), Madagascar, KX584888, KX584985. *Allophylusbicruris* Radlk., Barthelat 828 (MO), Mayotte, KX584889, KX584986. *Allophylusbojerianus* (Cambess.) Blume, Ratovoson 961 (MO), Madagascar, KX584890, KX584987. *Allophyluschaunostachys* Gilg, Mwangoko 729 (MO), Tanzania, KX584891, KX584988. *Allophyluschirindensis* Baker f., Hizza 26 (MO), Tanzania, KX584892, KX584989. *Allophyluscominia* Sw., Acevedo-Rodríguez 12216 (US), Mexico, KX584893, KX584990. *Allophyluscrassinervis* Radlk., Acevedo-Rodríguez s.n. (no voucher), Puerto Rico, KX584894, KX584991. *Allophylusdecipiens* (E. Mey.) Radlk., Phillipson 4194 (MO), South Africa, KX584895, KX584992. *Allophylusgardineri* Summerh., Pignal 1834 (MO), Mayotte, KX584897, KX584994. *Allophylushirtellus* (Hook. f.) Radlk., Cheek 5059 (?), KX584898, KX584995. *Allophyluspervillei* Blume, Hoffmann 399 (MO), Mayotte, KX584899, KX584996. *Allophyluspoungouensis* Pellegr., McPherson 16109 (MO), Gabon, KX584900, KX584997. *Allophyluspuberulus* (Cambess.) Radlk., Somner 1069 (US), Brazil, KX584901, KX584998. *Allophylusracemosus* Sw., Acevedo-Rodríguez 12180 (US), Mexico, KX584902, KX584999. *Allophylusrubifolius* (A. Rich.) Engl., Kuchar 23357 (MO), Tanzania, KX584903, KX585000. *Allophylus* sp., Acevedo-Rodríguez 14847 (NY), Brazil, KX584904, KX585001. *Athyanaweinmanniifolia* (Griseb.) Radlk., Acevedo-Rodríguez 11166 (US), Bolivia, KX584906, KX585003. *Balsasguerrerensis* Cruz Durán & K. Vega, Vega Flores 1318 (US), Mexico, KX584908, KX585005. *Bridgesiaincisifolia* Cambess., Landrum 9824 (NY), Chile, KX584909, KX585006. *Cardiospermumcorindum* L., Harder & Bringham 3495 (MO), Zambia, KX584912, KX585007. *Cardiospermumcuchujaquense* Ferrucci & Acev.-Rodr., Van Devender 92-1012 (ARIZ), Mexico, KX584914, KX585008. *Cardiospermumgrandiflorum* Sw., ATBP 603 (MO), Uganda, KX584915, KX585009. *Cardiospermumgrandiflorum* Sw., Gildenhuys H1 (?), Hawaii, KM062277, KM062362. *Cardiospermumheringeri* Ferrucci, Urdampilleta 437 (US), Brazil, KX584917, KX585010. *Cardiospermumurvilleoides* (Radlk.) Ferrucci, Urdampilleta 425 (US), Brazil, KX584922, KX585013. *Chimborazoalachnocarpa* (Radlk.) H.T. Beck, Wiggens 11060 (US), Ecuador, KX584923, KX585014. *Diatenopteryxsorbifolia* Radlk., Zardini 43371 (MO), Paraguay, EU720534, EU721303. *Dictyoneuraobtusa* Blume, Edwards KE142 (JCT), Australia, EU720428, EU721187. *Diploglottiscampbellii* Cheel, Chase 2048 (K), Australia, EU720457, EU721224. *Guindiliadissecta* (Covas & Burkart) Hunz., Ferrucci 2928 (CTES), Argentina, KX584926, KX585017. *Guioavillosa* Radlk., McPherson 18040 (MO), New Caledonia, EU720544, EU721314. *Haplocoeluminoploeum* Radlk., Lap 117 (?), FJ514259, FJ514265. *Houssayanthusbiternatus* (Weath) Rzed. & Calderón, Catalán & Terán 837 (MO), Mexico, KX584927, KX585018. *Houssayanthusincanus* (Radlk.) Ferrucci, Ferrucci 2710 (CTES), Argentina, KX584928, KX585019. *Jagerajavanica* (Blume) Kalkman, Chase 2130 (K), Bogor, EU721236, EU720468. *Lepisanthessenegalensis* (Poir.) Leenh., Callmander 627 (MO), Madagascar, EU720492, U72126. *Lophostigmaplumosum* Radlk., Acevedo-Rodríguez 6554 (US), Bolivia, KX584929, KX585020. *Macphersoniagracilis* O. Hoffm., Rabenantoandro 1081 (MO), Madagascar, EU720550, EU721320. *Mataybaguianensis* Aubl., Acevedo-Rodríguez 12342 (US), French Guiana, EU720527, EU721294. *Melicoccuslepidopetalus* Radlk., Acevedo-Rodríguez 11128 (US), Bolivia, EU720443, EU721206. *Paulliniaclathrata* Radlk., Acevedo-Rodríguez 14305 (US), Peru, KX584930, KX585021. *Paulliniacoriacea* Casar., Somner 1070 (RBR), Brazil, KX584931, KX585022. *Paulliniacuneata* Radlk., Acevedo-Rodríguez 14255 (US), Peru, KX584932, KX585023. *Paulliniaelegans* Cambess., Acevedo-Rodríguez 14976 (US), Brazil, KX584933, KX585024. *Paulliniahystrix* Radlk., Acevedo-Rodríguez 14417 (US), Peru, KX584934, KX585025. *Paulliniaimberbis* Radlk., Schunke Vigo 14928 (US), Peru, KX584935, KX585026. *Paulliniaolivacea* Radlk., Schunke Vigo 16002 (US), Peru, KX584936, KX585027. *Paulliniapinnata* L., Acevedo-Rodríguez 11088 (US), French Guiana, KX584937, KX585028. *Paulliniaprevostiana* Acev.-Rodr., Acevedo-Rodríguez 11113 (US), French Guiana, KX584938, KX585029. *Paulliniarubiginosa* Cambess., Thomas 12995 (US), Brazil, KX584939, KX585030. *Paulliniaspicata* Benth., Acevedo-Rodríguez 12344 (US), French Guiana, KX584941, KX585032. *Paulliniastellata* Radlk., Acevedo-Rodríguez 14958 (US), Brazil, KX584942, KX585033. *Paulliniaxestophylla* Radlk., Hoffman 5955 (US), Suriname, KX584943, KX585034. *Plagioscyphusunijugatus* Capuron, Buerki 145 (NEU), Madagascar, EU720475, EU721245. *Sapindusoligophyllus* Merr. & Chun, How 70627 (US), China, KX584944, KX585035. *Serjaniaaltissima* (Poepp.) Radlk., Acevedo-Rodríguez 14953 (US), Brazil, KX584945, KX585036. *Serjaniaampelopsis* Planch. & Lind., Acevedo-Rodríguez 11181 (US), Bolivia, KX584946, KX585037. *Serjaniacaracasana* (Jacq.) Willd., Acevedo-Rodríguez 15107 (US), Mexico, KX584947, KX585038. Serjaniacf.caracasana (Jacq.) Willd., Acevedo-Rodríguez 3483 (US), Guyana, KX584948, KX585039. *Serjaniaclematidifolia* Cambess., Somner 1078 (RBR), Brazil, KX584949, KX585040. *Serjaniacommunis* Cambess., Somner 1334 (US), Brazil, KX584950, KX585041. *Serjaniacuspidata* Cambess., Somner 1400 (US), Brazil, KX584951, KX585042. *Serjaniaemarginata* Kunth, Acevedo-Rodríguez 15135 (US), Mexico, KX584954, KX585043. *Serjaniaerythrocaulis* Acev.-Rodr. & Somner, Acevedo-Rodríguez 3729 (US), Brazil, KX584955, KX585044. *Serjaniaeucardia* Radlk., Somner 1072 (RBR), Brazil, KX584956, KX585045. *Serjaniafuscifolia* Radlk., Somner 1455 (RBR), Brazil, KX584957, KX585046. *Serjaniaichthyoctona* Radlk., Somner 1081 (RBR), Brazil, KX584960, KX585048. *Serjanialethalis* St. Hil., Roque 1860 (ALCB), Brazil, KX584961, KX585049. *Serjanialethalis* St. Hil., Somner 1381 (RBR), Brazil, KX584962, KX585050. *Serjaniamarginata* Casar., Acevedo-Rodríguez 11131 (US), Bolivia, KX584963, KX585051. *Serjaniamexicana* (L.) Willd., Acevedo-

Rodríguez 12014 (US), Jamaica, KX584965, KX585052. *Serjaniamexicana* (L.) Willd., Acevedo-Rodríguez 15080 (US), Mexico, KX584966, KX585053. *Serjaniapaniculata* Kunth, Acevedo-Rodríguez 15143 (US), Mexico, KX584967, KX585054. *Serjaniaperulacea* Radlk., Acevedo-Rodríguez 11134 (US), Bolivia, KX584968, KX585055. *Serjaniaunguiculata* Radlk., Acevedo-Rodríguez 15081 (US), Mexico, KX584969, KX585056. *Serjaniayucatanensis* Standl., Acevedo-Rodríguez 12183 (US), Mexico, KX584970, KX585057. *Talisianervosa* Radlk., Pennington 628 (MO), ~, EU720474, EU721244. *Talisiaobovata* A.C. Sm., Lombello 13 (MO), Brazil, EU720485, EU721255. *Thinouiacazumbensis* sp. nov., Medeiros 3401 (RB) Brazil, MT853074, MT847016. *Thinouiamucronata* Radlk., Keller 6919 (US), Argentina, KX584971, KX585058. Thinouiacf.mucronata Radlk., Medeiros 3800 (RB) Brazil, MT853076, MT847018. *Thinouiamyriantha* Radlk., Torke 2024 (HSTM), Brazil, MT853071, MT847013. *Thinouiaobliqua* Radlk., Medeiros 3793 (RB) Brazil, MT853075, MT847017. *Thinouia* sp., Medeiros 2193 (RB), Brazil, MT853072, MT847014. *Thinouiarestingae* Ferrucci & Somner, Somner 1074 (RBR), Brazil, KX584972, KX585060. *Thinouiatrifoliata* (Radlk.) Acev.-Rodr. & Ferrucci, Medeiros 3331 (RB), Brazil, MT853073, MT847015. *Thouiniaacuminata* S. Watson, Liston 633-2, ––, EU720478, EU721249. *Thouiniavillosa* DC., Hall 825 (US), Mexico, KX584975, KX585062. *Tristiropsisacutangula* Radlk., Chase 1358 (K), Bogor, EU720453, EU721220. *Urvilleachacoensis* Hunz., Acevedo-Rodríguez 11133 (US), Bolivia, KX584976, KX585063. *Urvilleachacoensis* Hunz., Keller 6834 (US), Argentina, KX584977, KX585064. *Urvilleapterocarpa* (Radlk.) Acev.-Rodr. & Ferrucci, Urdampilleta 321 (US), Brazil, KX585012, KX584921. *Urvillearufescens* Cambess., Somner 1073 (RBR), Brazil, KX584978, KX585065. *Urvilleaulmacea* Kunth, Acevedo-Rodríguez 15145 (US), Mexico, KX584979, KX585066. *Urvilleaulmacea* Kunth, Reyes-García 5585 (MO), Mexico, KX584980, KX585067. *Vouaranaguianensis* Aubl., Acevedo-Rodríguez 5031 (US), French Guiana, KX584981, KX585068.
